# Tympanitic Resonance in Pleuritis

**Published:** 1877-03

**Authors:** E. Fletcher Ingals

**Affiliations:** Chicago


### TYMPANITIC RESONANCE IN PLEURITIS.

By E. FLETCHER INGALS, M.D., Chicago.

In pleuritic effusions of considerable extent, the resonance over the anterior-superior portion of the affected side of the chest often has a tympanitic quality. Although most writers on diseases of the chest and physical diagnosis mention this, they omit to give a satisfactory explanation for the phenomenon.

In a review of the fourth volume of Ziemssen's Cyclopædia, in the February number of this Journal, we find an explanation of this sign by Fraentzel which we have not seen before. He holds that decrease in tension of the lung lowers, while decrease in volume raises the pitch of the percussion note; therefore he reasons that the sign in question is due to a relatively greater decrease in the tension than in the volume of the compressed lung. The tympanitic sound being considered of low pitch.

This is an ingenious theory but it is not satisfactory. At a temperature far below that of the human body water vapor is constantly rising, and at a temperature of 98½° F. water evaporates rapidly under a pressure of fifteen pounds to the square inch, even though removed from the favoring influences of a changing atmosphere. To verify this, fill a test tube, and invert in water heated to 98½° F.; very soon vapor will collect above the column of water in the tube. If the heat be maintained we can easily collect a considerable quantity of vapor. If a funnel be attached to the tube, so as to collect the bubbles from a larger surface, the vapor will be obtained more rapidly.

To make the conditions of the water in the test tube conform, as nearly as possible, to those of fluid in the pleural sac, we may attach to the tube an elastic rubber bag, having both filled with water. By maintaining a temperature equal to that of the body, we will obtain the same results as in the first experiment.

In these experiments there will be as much pressure upon fluid in the tube as upon that contained in the chest in pleuritic effusions; at least until the pleural sac becomes distended, and in most cases when distension is great, there is a corresponding elevation of temperature in the body, which increases the tension of the watery vapor sufficiently to overcome the augmented pressure.

These experiments seem to leave no opportunity for a reasonable doubt that evaporation takes place from pleuritic effusions.

It seems fair to presume that alterations in the pleura, either as the direct result of inflammation, or as a consequence of pressure, which are sufficient to prevent the absorption of fluid, must likewise hinder the absorption of watery vapor; therefore, notwithstanding the influence which volume and tension of the lung, or air in the larger bronchi, may have in the production of tympanitic resonance, we believe the sign under consideration is in great part due to a collection of vapor above the fluid in the pleural cavity.

